# Targeted transcriptomic analyses of tuberculosis treatment response and outcomes in The Gambia

**DOI:** 10.3389/ftubr.2025.1540194

**Published:** 2025-09-02

**Authors:** Ismaila L. Manneh, Fatoumatta Darboe, Haddijatou Jobe, Binta Sarr Kuyateh, Ousainou Cham, Olumuyiwa Owolabi, Brezesky Kotanmi, Andrea Rachow, Salome Charalambous, Kathrin Held, Hazel M. Dockrell, Jayne S. Sutherland

**Affiliations:** ^1^Vaccines and Immunity Theme, Medical Research Council Unit The Gambia at London School of Hygiene and Tropical Medicine, Fajara, Gambia; ^2^Division of Experimental Medicine, University of California, San Francisco, San Francisco, CA, United States; ^3^Statistics and Bioinformatics, Medical Research Council Unit The Gambia at London School of Hygiene and Tropical Medicine, Fajara, Gambia; ^4^Institute of Infectious Diseases and Tropical Medicine, Ludwig Maximilian University of Munich University Hospital, Munich, Germany; ^5^The Aurum Institute, Johannesburg, South Africa; ^6^German Centre for Infection Research (DZIF), Partner Site Munich, Braunschweig, Munich, Germany; ^7^Fraunhofer Institute for Translational Medicine and Pharmacology, Immunology, Infection and Pandemic Research, Munich, Germany; ^8^Unit Global Health, Helmholtz Zentrum München, German Research Centre for Environmental Health (HMGU), Neuherberg, Germany; ^9^Department of Infection Biology, London School of Hygiene and Tropical Medicine, London, United Kingdom

**Keywords:** *Mycobacterium tuberculosis* disease, treatment response, treatment outcome, tuberculosis, gene expression

## Abstract

**Background:**

Despite availability of effective treatment regimens for drug-susceptible Tuberculosis (TB), some patients still experience poor treatment outcomes. Currently tools for monitoring treatment outcomes are dependent on detection of mycobacteria in sputum, which are slow, expensive and poor at predicting relapse and failure. This study aims to identify new blood-derived markers for predicting treatment response and outcomes.

**Methods:**

Whole blood was collected in PAXgene tubes from patients with microbiologically confirmed TB at diagnosis, week 2, and at months 2, 4, and 6. Treatment response and outcomes were determined by culture and gene expression was compared between slow and fast responders; and between patients with good (cured) and poor treatment outcomes (failure and recurrent TB) using targeted RNA gene expression. Gene signatures were developed using random forest classification models.

**Results:**

Significant changes in gene expression were detected over the course of the TB treatment. Notably, major gene expression differences were observed at diagnosis between subsequently cured patients and patients who experienced poor treatment outcomes while minimal changes were detected between slow and fast responders among cured patients at diagnosis. A 7-gene end of treatment signature distinguished patients with good outcomes from those with poor treatment outcomes with area under the curve (AUC) of 0.91 (95% CI 0.85–0.99), 0.98 (95% CI 0.96–0.99), and 1.0 (95% CI 0.99–1.00), at baseline, month 2 and 6, respectively. Additionally, a 6-gene month 2 signature discriminates slow from fast responders with AUCs of 0.49 (95% CI 0.33–0.64), 0.58 (95% CI 0.07–1.00), and 0.93 (95% CI 0.78–1.00) at diagnosis, week 2 and month 2, respectively.

**Conclusion:**

The study identified genes signatures associated with TB treatment response and outcomes suggesting potential utility for treatment monitoring.

## Introduction

Tuberculosis (TB) remains the leading cause of death from a single infectious disease, despite the availability of a vaccine and effective treatments (1). Timely diagnosis and treatment are paramount for optimal case management and to reduce transmission, mortality and the development of resistance (1, 2).

Standard treatment for drug-susceptible TB consists of a 6-month multi-drug regimen, beginning with a 2-month intensive phase using Isoniazid, rifampicin, pyrazinamide and ethambutol to kill highly replicating bacteria, followed by a 4-month continuation phase of Isoniazid and rifampicin to kill the dormant bacteria (2, 3). While successful treatment is achieved in most drug-susceptible TB patients, some patients do not achieve complete clearance of the bacteria and have an increased risk of relapse and treatment failure (4, 5). Furthermore, the prolonged duration of treatment is associated with significant challenges with treatment adherence, drug toxicity and the development of drug resistance (2, 6).

Current tools for monitoring treatment response are based on detection of the pathogen, *Mycobacterium tuberculosis* (Mtb) in patients sputum, using culture and smear microscopy (4, 7). However, both culture and smear microscopy have significant limitations. Culture is limited by long turnaround times, high costs, susceptibility to contamination and low accuracy in predicting relapse and treatment failure while smear microscopy has poor sensitivity (4). Moreover, these tests rely on quality sputum specimens, which can be difficult to obtain from people with paucibacillary disease such as children, people with HIV or those with extrapulmonary TB (4, 7). Consequently, new tools are needed for monitoring and predicting TB treatment response and outcome.

WHO has prioritized the search for cost-effective, user-friendly non-sputum tests for TB diagnosis and treatment response, establishing target product profiles for such tests (8–10). Several studies have reported host blood gene expression patterns capable of distinguishing TB patients from latently infected or healthy people underscoring the potential of blood as a suitable specimen for TB diagnosis (11–14). Some transcriptomic signatures including Sweeney3 (15), Risk6 (16), TB22 (17), and Thompson5 (18) exhibit dynamic changes during treatment that suggest potential for also monitoring treatment response.

Although blood-derived gene markers hold promise as surrogates for monitoring treatment response, few studies have specifically focused on identifying markers that differentiate between fast and slow treatment responders who are at greater risk of treatment failure or distinguish cured patients from those with unfavorable treatment outcomes. Additionally, many studies focus only on diagnostic and end-of-treatment timepoints, potentially overlooking critical gene expression changes that could explain the variability in treatment responses. Patients with poor outcomes, including treatment failure and relapse, require longer treatment regimens, increasing their risk of drug toxicity and contributing to transmission (6, 19). Identifying slow responders and patients at risk of poor outcomes early in treatment is essential for developing shorter, and more personalized regimens. Thus, we aimed to describe gene expression dynamics in blood of TB patients during treatment and to identify gene signatures that distinguish slow from fast responders; and between cured patients and those who experience treatment failure and recurrent TB.

## Materials and methods

### Ethical approval

The study was approved by the London School of Hygiene and Tropical Medicine (LSHTM) Ethics Committee and the Joint Gambia Government–MRC Unit Ethics Committee (LEO 21727).

### Study design

This study was nested within a prospective multi-center observational cohort study, TB Sequel, in which adult patients with microbiologically confirmed tuberculosis diagnosed using GeneXpert MTB/RIF Ultra assay (Cepheid, USA) were enrolled (20). Participants were followed up at week 2 and at months 2, 4, and 6 of treatment, during which sputum samples were collected for Mycobacteria Growth Indicator Tubes culture and whole blood was collected in PAXgene^®^ Blood RNA Tube (BD Bioscience, USA) for transcriptomic analyses. Patients were followed up for 18 months after treatment completion to confirm their TB treatment outcome. Participants included in this sub-study were selected based on their treatment response outcome and sample availability.

### Classifications

Patients were defined as microbiologically cured if they had a negative sputum culture upon completion of treatment and remained disease-free for at least 1-year after standard 6-month anti-tuberculosis treatment. Those with positive cultures at the end of 6-month treatment were deemed treatment failures. Patients who presented with clinical or microbiologically confirmed TB within 1 year of standard TB treatment completion despite having a negative culture at the end of treatment were classified as having recurrent TB, without distinguishing between relapse and reinfection. Patients were grouped into fast and slow treatment responders based on sputum culture conversion at month 2 of treatment. Fast responders were defined as those achieving negative cultures by month 2, while slow responders were defined as those that achieved negative cultures only after month 2.

### Sample collection and total RNA extraction

At diagnosis and at week 2, months 2, 4, and 6 of treatment, 2.5 ml of whole blood was collected from all study participants in a PAXgene tube and stored at −80°C until needed. RNA was extracted from selected participants using the PAXgene Blood RNA Kit (Qiagen, Switzerland) following manufacturer's instructions. The quality and concentration of the extracted RNA were assessed using Agilent ScreenTape (Agilent Technologies, Europe) and Qubit fluorometer (Thermo Fisher Scientific, Europe). RNA input for gene expression analyses was determined based on fragments of RNA >200 nucleotides (DV200) measured with Agilent ScreenTape.

### Gene expression analysis

Gene expression was analyzed using targeted RNA sequencing with nCounter host response panel (NanoString Technologies, USA). This panel included 12 housekeeping genes and 773 host response transcripts spanning 50 different pathways. A total of 100 ng of RNA was mixed with a Mastermix containing barcoded capture and reporter probe pairs specific to each transcript and hybridized for 16 h at 65 °C in a thermocycler. After hybridization, samples were transferred to the nCounter Prep-Station were unhybridized probes were removed and purified RNA was loaded onto an analysis cartridge. Quantitative measurement of hybridized RNA was obtained by scanning reporter probes on the cartridge using the nCounter digital Analyzer (NanoString Technologies, USA). Signal values were background subtracted and normalized to the most stable housekeeping probes, identified using the geNorm algorithm from the NormqPCR package (version 1.50) (21).

### Statistical analysis

Gene expression data was analyzed using ROSALIND^®^ (versionv3.16; https://rosalind.bio/). Differential expression was assessed using a negative binomial mixture model for low-expression genes and a simplified negative binomial model for high-expression genes, defined by a threshold set at 10 times the background noise. Significance was set at a fold change (FC) in expression of ≥1.5 and *p*-values ≤ 0.05, adjusted for false discovery rate (FDR) using the Benjamini–Hochberg method (22). When no differentially expressed genes (DEGs) were detected at this threshold, the cutoff was relaxed to an FC of ≥1.25 and an unadjusted *p*-value ≤ 0.05 to further explore gene expression differences. Cell type profiling was performed using immune cell typing module in nSolver (version 4.0). Functional analysis of DEGs was performed using nSolver and the database for annotation, visualization, and integrated discovery (DAVID) (23). Data was visualized using heatmaps and volcano plots generated using ComplexHeatmap (24), and ggplot2 (25) packages in R programming language (version 4.4.0).

Categorical patient characteristics were compared using Chi-square or Fisher's exact test, while continuous variables were analyzed using Wilcoxon rank-sum test in base R. Longitudinal analysis of gene expression during treatment were analyzed using the MaSigPro R package (version 1.76.0) (26). Random Forest classification models were constructed to identify gene signatures using the Caret R package (version 6.0-94) (27). Random Forest models were built using 500 iterations of 80/20 train-test splits, following preprocessing to remove near-zero variance and highly correlated features (cutoff = 0.8). In each iteration, models were trained with five-fold cross-validation using 500 trees, and performance was assessed using accuracy and AUC on the held-out test sets. Features appearing consistently (100%) in models achieving ≥70% accuracy were selected to construct a simplified Random Forest model. Model performance was evaluated using area under the receiver operating characteristic curve (ROC AUC) calculated using the pROC R package (version 1.18.5) (28).

## Results

### Patients demographic information

Samples from 69 microbiologically confirmed TB patients who completed standard 6-month anti-tuberculosis treatment were included in the study. Of these, 59 (85.5%) achieved cure, 7 (10%) experienced treatment failure and three patients (4.3%) had recurrent TB (Table 1). All cured patients exhibited culture conversion at the end of 6 months of treatment. Of the cured patients, 29 (49%) were identified as slow responders (culture positive at 2 months but negative by 6 months) while 30 (51%) were termed fast responders based on sputum culture conversion results at month 2 of treatment. There was no significant difference in the median ages of patients with treatment failure (44 years, IQR 40.5–52.5), recurrent TB (37 years, IQR 36–46.5) and cured patients (32 years, IQR 27.5–40). Most study subjects were males, constituting 62%, 71%, and 66% of cured, failure and recurrent TB patients, respectively. Among cured patients, two had a history of TB while one recurrent TB case was coinfected with HIV. There was no association between culture, GeneXpert Ultra result and GeneXpert grade with treatment response and outcomes.

**Table 1:** Patients' demographic characteristics.

**Characteristics**	**Cured *N* = 59**	**Treatment failure *N* = 7**	**Recurrent TB *N* = 3**	***p*-value**
**GeneXpert ultra positive**	57 (97)	7 (100)	3 (100)	>0.999
Trace	1 (2)	0 (0)	0 (0)	
Low	3 (5)	0 (0)	0 (0)	
Medium	15 (25)	3 (43)	1 (33)	
High	38 (64)	4 (57)	2 (66)	
**Responder status**				
Slow responder	29 (49)	3 (43)	1 (33)	>0.999
Fast responder	30 (51)	4 (57)	1 (33)	
Age, median (IQR)	32 (27.5–40)	44 (40.5–52.5)	37 (36–46.5)	0.045^a^
Male	43 (62%)	5 (71%)	2 (66%)	>0.999
HIV	0 (0)	0 (0)	1 (33)	0.043
Active smoker	6 (0)	2 (29)	0 (0)	0.327
TB History	2 (3.4)	0 (0)	0 (0)	>0.999

N, number of patients; Parenthesis, percentages; IQR, interquartile range; P value, Fisher exact test.

a Kruskal Wallis test.

### Gene expression changes were detected as early as week 2 of treatment

**Figure 1:**
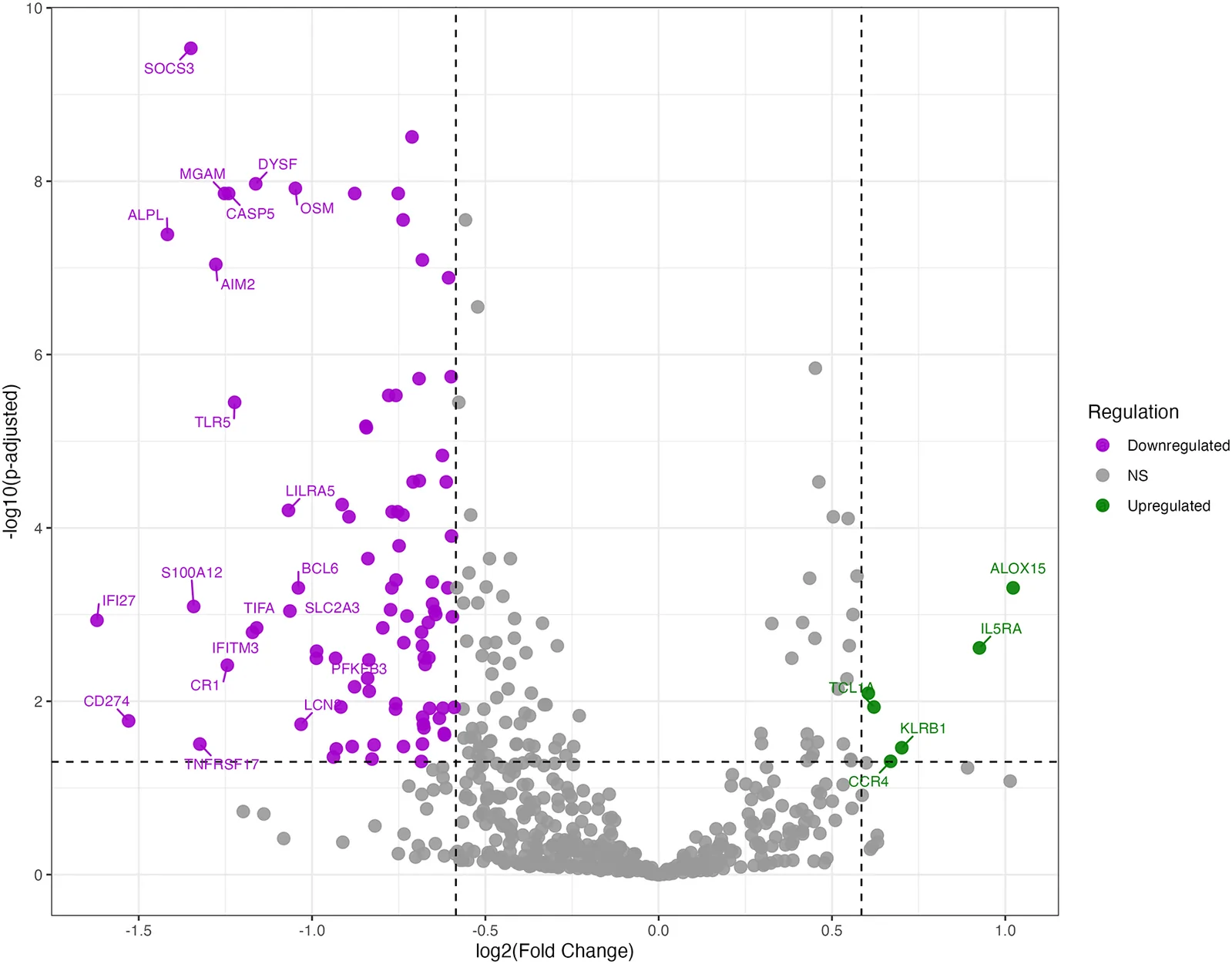
Differentially expressed genes at month 2 of TB treatment among cured patients. Volcano plot of DEGs at month 2 of treatment relative to baseline among cured patients. Plot displays the log_2_ fold changes (*x*-axis) against -log_10_ adjusted *p*-values (*y*-axis) for genes analyzed. The top 20 most differentially expressed genes are labeled. Horizontal dashed lines indicate the significance thresholds for adjusted *p*-values < 0.05, while vertical dashed lines represent the fold change thresholds of ±1.5 FC. Downregulated genes are shown in purple, and upregulated genes are shown in green.

**Figure 2:**
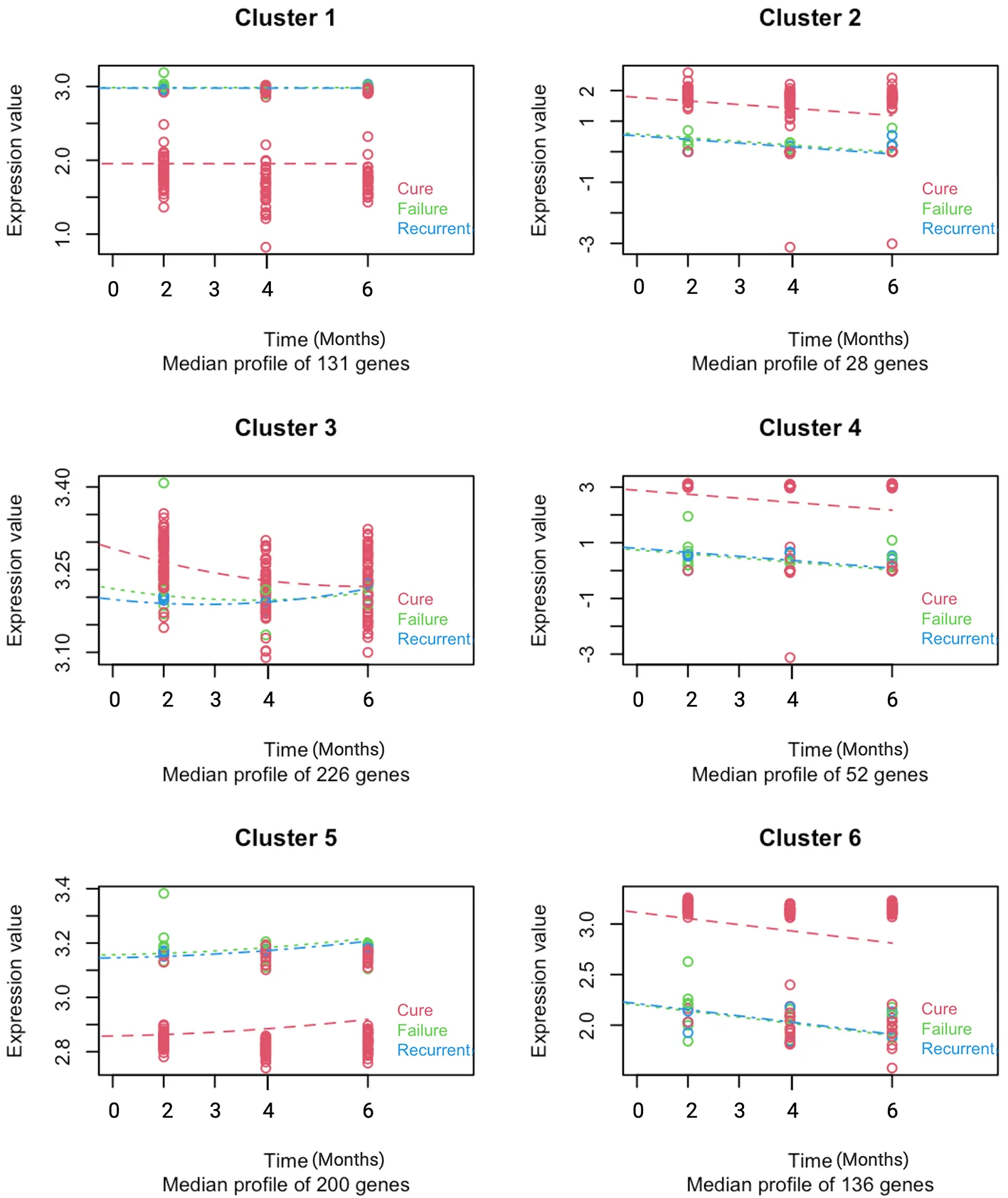
Distinct longitudinal gene expression patterns differentiate cured, recurrent, and treatment failure TB patients. MaSigPro identified six gene clusters with shared expression trajectories during treatment in patients who were cured, had recurrent TB, or experienced treatment failure. Gene expression data collected at baseline and months 2, 4, and 6 are plotted over time. Cured (*n* = 59) are shown in red, treatment failure (*n* = 7) in green, and recurrent TB (*n* = 3) in blue. Dots represent individual gene values; dashed lines show median expression per cluster at each time point.

To analyse changes in gene expression during treatment, we performed pairwise comparison of gene expression across treatment time points relative to baseline expression in cured, treatment failure and recurrent TB patients. Significant changes in gene expression were detected as early as week 2 and increased further with longer treatment duration. At week 2, and months 2, 4, and 6 a total of 23, 94, 161, and 175 genes were differentially expressed (DEGs) in cured patients, respectively. Figure 1 depicts differentially expressed genes detected at month 2. A similar pattern of expression changes was observed in patients with treatment failure and recurrent TB but with a smaller number of DEGs. A total of, 41, 31, and 27 DEGs were observed at months 2, 4, and 6, respectively in patients with treatment failure, while 6, 11, and 13 DEGs were detected at months 2, 4, and 6, respectively in recurrent TB patients (Supplementary Tables S1–S3). To capture the dynamic changes in gene expression during treatment, we used the maSigPro package (26) for time course analyses of longitudinal data collected from cured, failed and recurrent TB patients. maSigPro identified patterns in DEGs across the three datasets and grouped them into six clusters based on their patterns of expression over time (Figure 2; Supplementary Table S4). Clusters 2, 3, 4 and 6 were downregulated during treatment while genes in cluster 5 were upregulated. Cluster 1 genes did not vary much during treatment. The expression level of downregulated genes was relatively higher in cured patients than in treatment failure and recurrent TB patients, while the expressions of the relatively stable and upregulated genes were relatively higher in patients with treatment failure or recurrent TB. Notably, dysregulated clusters include genes involved in innate immune and inflammatory responses (Supplementary Figure S1), suggesting that variation in host immune response to Mtb infection may influence treatment outcomes.

### Comparison of gene expression profiles between slow and fast treatment responders

**Figure 3:**
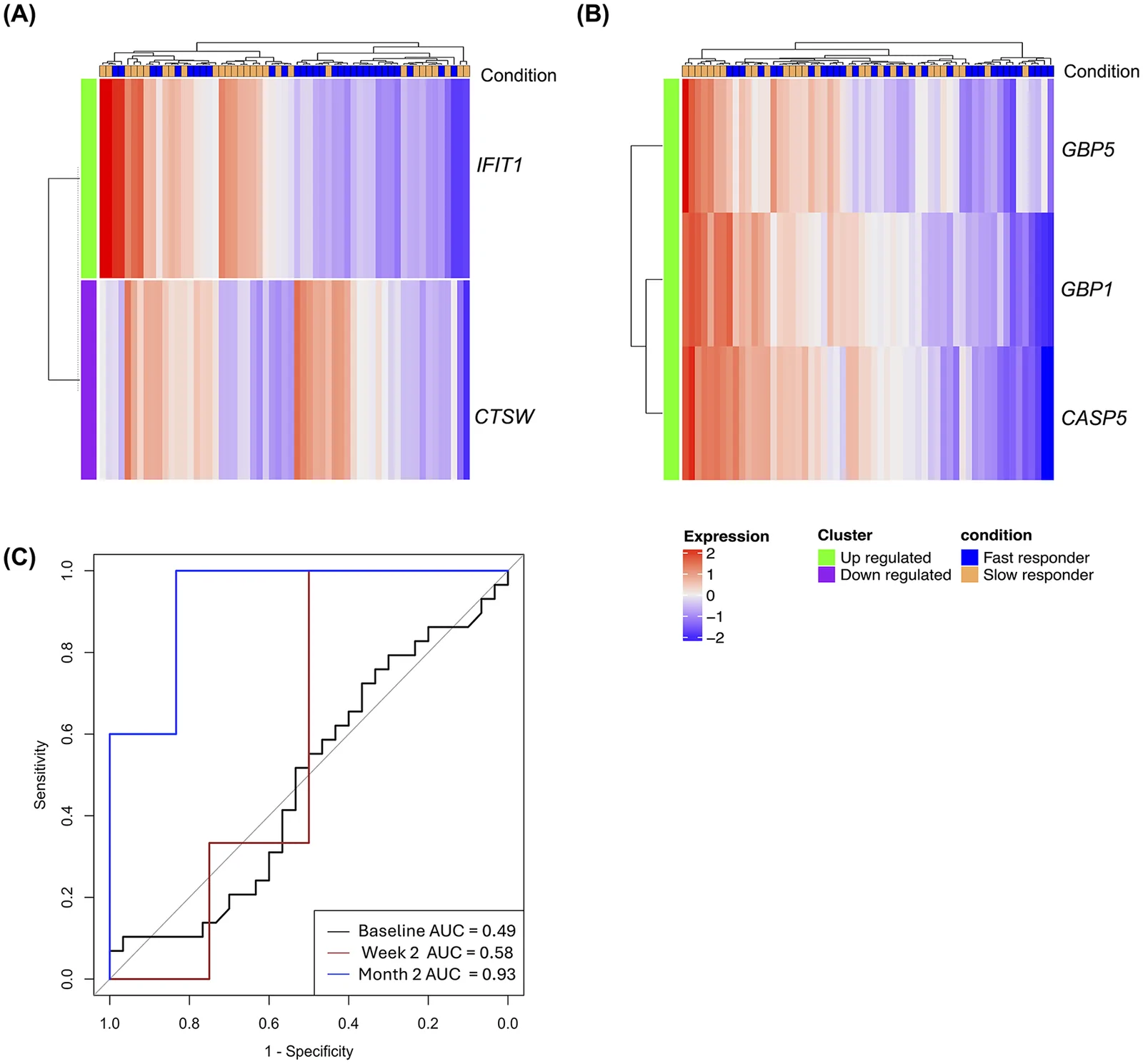
Early treatment gene expression signatures distinguish fast from slow treatment responders. **(A)** Heatmap of DEGs between slow (*n* = 29) and fast responders (*n* = 30) at diagnosis. **(B)** Heatmap of DEGs between the two groups at month 2 of treatment. For these explorative analyses, cut off for DEGs was set at *p* value ≤ 0.05 and a fold change of 1.25. Green labeled rows represent upregulated genes; purple rows represent downregulated genes; blue labeled columns are slow responder and orange labeled column are fast responders. **(C)** ROC curves with AUC values for a six-transcript signature distinguishing between slow and fast responders at baseline, week 2, and month 2.

When gene expression levels were compared at baseline, week 2 and month 2 between slow and fast treatment responders, no DEGs were detected that met a predefined stringent criterion of FDR < 0.05 and >± 1.5-fold. However, upon relaxation of this criteria to a fold difference of ≥1.25 and unadjusted *p*-value ≤ 0.05, two DEGs were observed, with one gene upregulated (*IFIT1*), and one gene downregulated (*CTSW*) in slow responders at diagnosis (Figure 3A). Similarly, application of these criteria at week 2 and month 2, yielded one DEG (*TCLA1*) and three DEGs (*GBP5, GBP1*, and *CASP5*), respectively (Figure 3B; Supplementary Table S5). However, these genes were individually poor predictors of slow and fast responders (AUC ≤ 0.50) at their respective timepoints. Random forest modeling to identify the optimal combinations of genes that distinguished slow from fast responders resulted in a 6-gene signature (*BCR, GNLY, IL11RA, KLRC1, MAP3K7*, and *TBK1)* derived from month 2 data that discriminated between the two groups with an AUC of 0.93 (95% CI 0.78–1.00; Figure 3C). The model failed to predict slow from fast responders at baseline and week 2, attaining AUCs of 0.49 (95% CI 0.33–0.64) and 0.58 (95% CI 0.07–1.00), respectively.

### Cured patients showed distinct expression profile from patients with poor treatment outcomes at diagnosis

**Figure 4:**
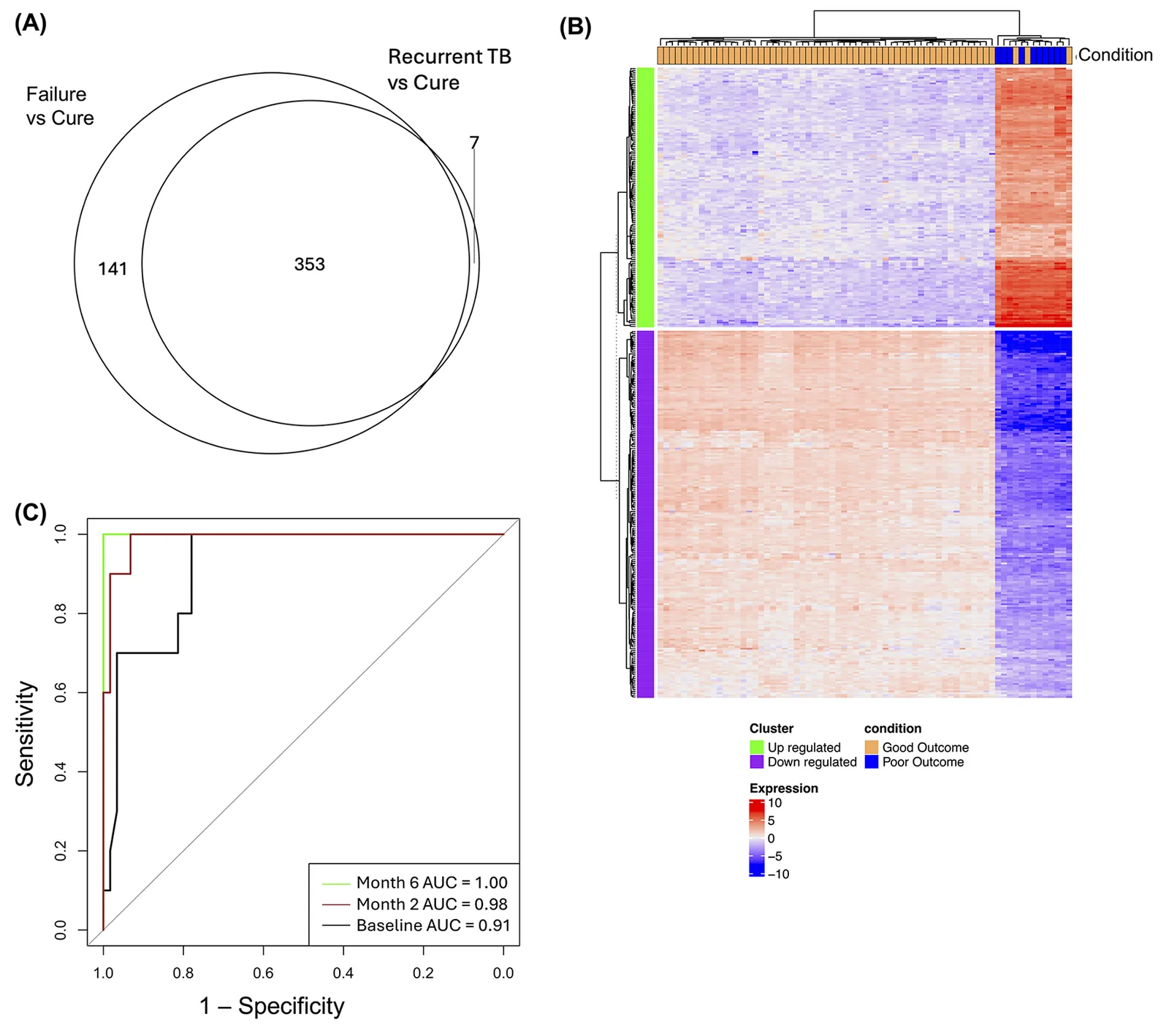
Gene expression signatures distinguish good and poor treatment outcomes. **(A)** Venn diagram illustrating overlapping DEGs in treatment failure and recurrent TB relative to cured patients at month 6 of treatment. **(B)** Heatmap showing DEGs between patients with good treatment outcomes (cured; *n* = 59) and those with poor outcomes (treatment failure and recurrent TB; *n* = 10) at baseline. Green labeled rows are upregulated genes; purple rows are downregulated genes; orange labeled column are patients with good treatment outcome and blue column are patient with poor outcomes **(C)** ROC curves with AUC values for transcript set that distinguish between good and poor treatment outcomes at baseline, month 2, and month 6.

Comparative analyses of gene expression profiles of cured patients and patients who experienced treatment failure at the end of treatment yielded a total of 494 DEGs of which 198 genes were upregulated and 296 downregulated. Similarly, a total of 360 DEGs were found in recurrent TB when compared to cured patients, comprising 99 upregulated and 261 downregulated genes (Figure 4A; Supplementary Table S6). Gene pathway analysis using KEGG pathways underscores the critical role of cellular signaling and receptor-mediated interactions, particularly chemokine and cytokine signaling, JAK-STAT signaling, and IL-17 signaling pathways, in the shared differentially expressed genes (DEGs) associated with treatment failure and recurrent TB (Supplementary Figure S2). Interestingly, we found no differentially expressed genes between patients with recurrent TB and those with treatment failure at diagnosis. Due to the similarity in gene expression profiles observed between treatment failure and recurrent TB patients, we pragmatically combined these two outcomes into a single group termed “poor outcome,” in contrast to cured patients, who were categorized as having a “good outcome” (Figure 4B). Using random forest to identify optimal gene combinations that classify good treatment from poor treatment outcomes, a 7-gene signature was identified that perfectly discriminated between the two outcomes at end of treatment [AUC 1.0 (95% CI 0.99–1.00)] (Figure 4C). This gene panel consist of *CCR6, CTSW, GADD45B, GZMH, LEF1, MARCKS*, and *NLRC4*. The signature predicted poor from good treatment outcome at baseline and month 2 with AUCs of 0.91 (95% CI 0.85–0.99), and 0.98 (95% CI 0.96–0.99), respectively.

### Cell-type profiling reveals a relative abundance of T-helper 1 (Th1) related genes in cured TB patients

**Figure 5:**
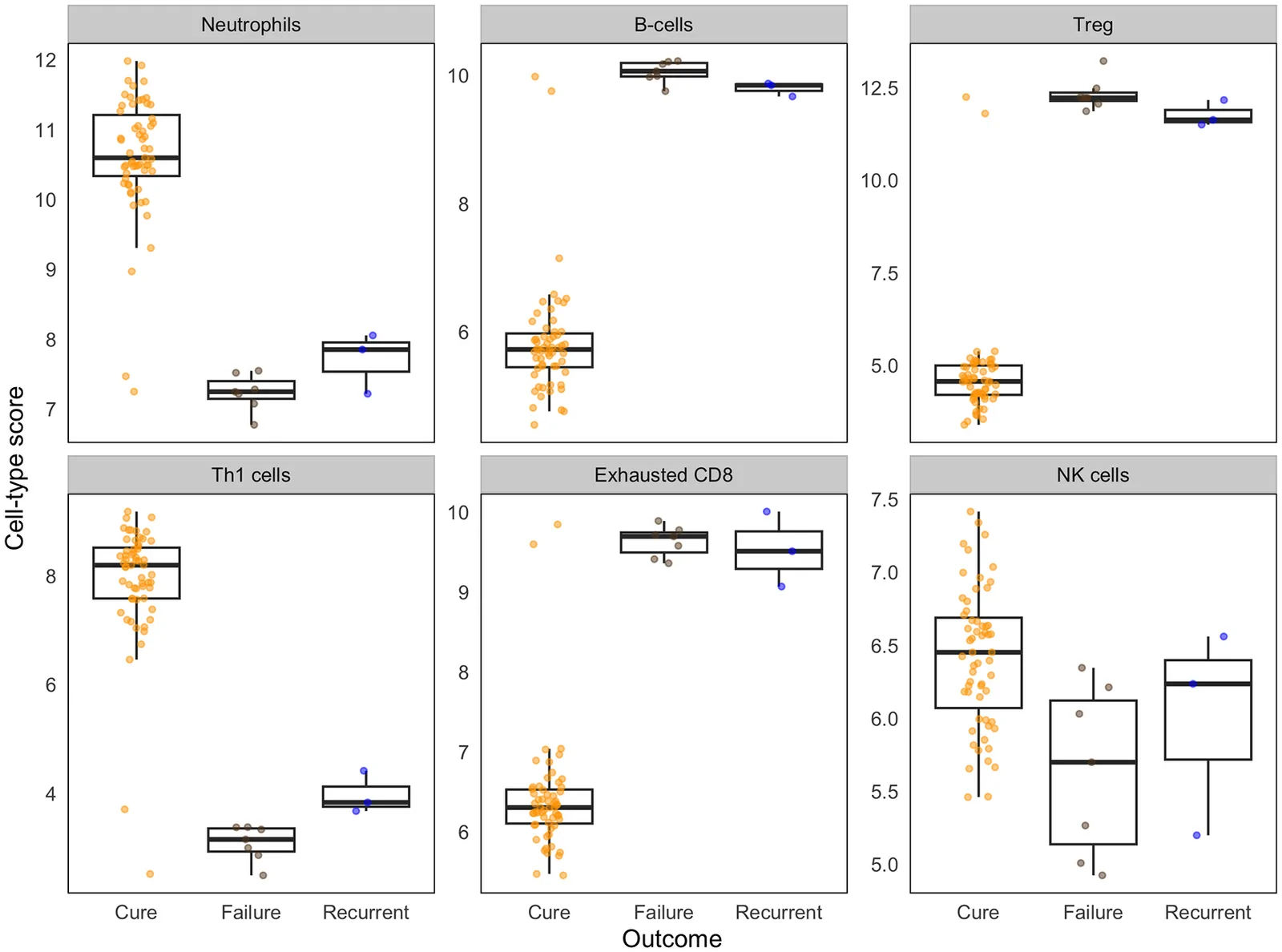
Differences in baseline immune cell-type scores associate with treatment outcomes. Cell-type scores were calculated based on the relative expression of cell type-specific genes using NanoString cell-type modules. Box plots show the median and interquartile range of cell type specific genes, with individual data points overlaid. Data are shown for cured patients (*n* = 59), treatment failure (*n* = 7), and recurrent TB (*n* = 3).

Relative cell-type abundance was determined from the gene scores of DEGs calculated using NanoString's Cell type profiling module as previously described (29). Cell type analyses showed relative expression of leucocyte subsets between groups including T cells, B cells, macrophages, cytotoxic cells, neutrophils, B cells, and NK cells based on expression of cell-type specific markers. Patients with good outcomes showed a relative higher cell expression score for CD4 Th1 profile *(TBX21)* and neutrophil genes *(CEACAM3, CSF3R, FCAR, FCGR3A/B, FPR1, S100A12*, and *SIGLEC5)* and NK cell genes *(NCR1, XCL1/2)* while poor treatment outcome was associated with relatively higher expression of Treg *(FOXP3)*, B cell *(BLK, CD19, FAM30A, FCRL2, MS4A1, PNOC, SPIB, TCL1A*, and *TNFRSF17)* and exhausted CD8 T cell genes *(CD244, EOMES*, and *LAG3*; Figure 5).

## Discussion

Developing new non-sputum-based tools for monitoring TB treatment is a critical priority, as current sputum-based methods which rely on Mtb detection have significant limitations including poor sensitivity (smear) and long turnaround times (culture) (4, 7). In this study, we explored the potential of blood host transcriptomics for monitoring treatment response through targeted RNA gene expression profiling. We identified gene signatures associated with slow treatment response and poor outcomes suggesting the possibility of providing personalized treatment regimens that could improve treatment success.

Previous studies have shown that detectable gene expression changes occur early in the course of anti-TB treatment, particularly by week 2 (30, 31). However, in this study, we observed an incremental increase in the number of differentially expressed genes (DEGs) over time, with significant changes already detectable at week 2 and continuing through subsequent treatment timepoints. These differences compared to previous studies are likely because we used a targeted sequencing approach which analyses a defined set of host-response genes, rather than providing bulk analysis of all genes. Notably, the expression levels of upregulated genes were relatively higher in patients with treatment failure and recurrent TB suggesting ongoing inflammation, while downregulated gene clusters were relatively more highly expressed in patients who were cured. These dysregulated clusters were enriched in genes involved in innate immune responses to pathogens and inflammation, suggesting that differences in the innate immune response to Mtb infection may contribute to treatment outcomes.

We identified a combination of six genes, which accurately classified slow responders from fast responders at month 2 with an AUC of 0.93 (95% CI 0.78–1.00) at month 2, demonstrating potential value for treatment monitoring. However, its predictive accuracy at earlier timepoints was poor. The genes in this signature, *BCR, GNLY, IL11RA, KLRC1, MAP3K7*, and *TBK1* are involved in innate immune signaling pathways including host-pathogen interactions, again suggesting that differences in innate immune response to infection may contribute to the short-term treatment outcome. Slow responders have higher risk of poor outcomes following treatment (19). Therefore, early identification of high-risk patients during treatment could facilitate the development of more personalized treatment strategies (32). The limited differences in gene expression between slow and fast responders observed in our study may be partly due to the relatively low number of target genes analyzed. Previous studies have reported significant differences in various cytokines and acute phase proteins such as IL-8, IL-1ra, CRP, IL-2Rα, VEGFR2, and MCP-3 between slow and fast treatment responders (33). However, we did not detect these differences at the transcriptional level, which may suggest the involvement of post-transcriptional regulation or other molecular mechanisms. While a fast response to therapy is currently the best surrogate for early treatment success, findings from trials involving shorter anti-tuberculosis regimens have revealed that early conversion does not eliminate the risk of relapse (34). Moreover, it is unclear if early conversion among cured patients correlates with improved lung function following treatment.

Our results show large expression differences between cured patients and those with treatment failure and recurrent TB disease at end of treatment. Dysregulated genes in patients with treatment failure and recurrent TB compared to those who were cured, show increased expression of chemokine signaling, IL-17 signaling, and JAK-STAT signaling pathways which play key roles in immune regulation during TB. IL-17 and chemokine signaling are associated with neutrophil recruitment and inflammation (35), while JAK-STAT signaling is involved in interferon responses (36). The heightened activity of these pathways may reflect persistent or unresolved inflammation, potentially contributing to poor treatment outcomes. From a clinical perspective, small signatures are more desirable for patient stratification as they are easier to translate into rapid diagnostic applications. Using random forest, we identified a 7-gene signature that accurately discriminates between patients with good and poor treatment outcomes. However, this finding should be validated in a larger and more diverse cohort to assess for robustness and generalizability. There have been some studies that identified gene signatures that adequately predict treatment response at initiation of treatment. A 10-gene signature was recently described that adequately predicted relapse at diagnosis with an AUC of 0.85 but was poor at predicting treatment failure in the discovery dataset (37). Two prognostic signatures, RISK6 (16) and RESPONSE5 (18), described in South African TB patients showed good prediction for treatment failure at the end of treatment with an AUC of 0.88 and 0.99%, respectively. Future studies should explore the combination of transcriptomic biomarkers and other markers such as proteomics, metabolomics, cellular markers and patients' clinical presentations for better biosignatures of treatment outcomes.

Cell type analyses using NanoString immune cell type profiling (29) revealed a significant difference in relative cell type abundance in T cells between patients with good and poor treatment outcomes. For example, we saw a relatively higher expression score for Th1 cells and Neutrophil related genes in cured patients suggesting the on-going inflammation in poor outcome is not related to these cell types. Tuberculosis has been associated with a neutrophil-driven gene expression profile (38) and Th1 cells produce pro-inflammatory cytokines such as IFN-γ, TNF, and IL-1 which activate the antimycobacterial function of macrophages (39, 40). Previous studies have found lower expression of Th1 associated genes and other adaptive immune system effectors cells in peripheral blood of active TB patients compared to healthy controls, associating it with migration of immune effector cells to the lung tissue in response to infection (41, 42). Therefore, the relatively higher Th1 gene expression in cured patients may suggest a better capacity to mount a protective Th1 immune response in this group. Our data also show that poor treatment outcome was associated with higher relative expression of exhausted CD8, Tregs, and B cell profiles. This may suggest a reduced effector functionalities of CD8 cells and a dampened immune response in patients with poor treatment outcomes, potentially favoring bacteria persistence. Future studies should investigate this hypothesis with flow cytometry or single cell sequencing which was not possible in the current study.

Our study found very little gene expression differences between patients with recurrent TB and those with treatment failure. The few dysregulated genes detected were genes involved in antigen recognition and immune response and suggests some differences in innate immunity response. However, due to the small number of patients in our study, we were unable to determine if this difference alone could influence treatment outcome. Although whole genome sequencing was performed to identify TB strains from all study participants, the small number of recurrent TB cases was insufficient to allow stratification into relapse and reinfection for any meaningful comparisons.

The study was limited by the small number of patients with poor treatment outcomes which limited our ability to validate signature models using a holdout validation set. As poor treatment outcomes are rare, future studies should consider pooling together biospecimens from different cohorts to overcome this challenge. Computational approaches have demonstrated the feasibility of developing TB signatures using multiple datasets from different studies and populations for both diagnosis (15) and treatment response monitoring (43). While targeted RNA sequencing may overlook some potentially important genes in a broader discovery effort, it offers significant advantages in signature validation, including cost-effectiveness, increased sensitivity and more streamlined data processing (44). Future research should focus on validating gene signatures identified in this and similar studies using a targeted sequencing approach, leveraging custom gene panels for more precise and focused evaluations.

In summary, our study identified sets of genes that adequately predict treatment outcomes in our cohort early in during treatment. The diagnostic utility of these gene signatures should now be validated in a different and larger cohort. A prognostic test that can effectively stratify TB patients at risk of poor treatment outcomes would greatly impact TB treatments. Given the relatively low occurrence of treatment failure and relapse in most TB studies, future studies should consider integrating samples from different cohorts to increase sample size, diversity, and generalizability of study findings.

## Data Availability

The original contributions presented in the study are included in the article/Supplementary material. The dataset analyzed for this study can be found in Gene expression Omnibus (GEO) (Accession number GSE295312).

## Supplementary material

Supplementary Figure S1 Functional analyses of differentially expressed genes during treatment. Biological pathways enriched in dysregulated genes during treatment. a) Top 10 biological processes enriched in downregulated genes. b) Top 10 biological processes enriched in downregulated genes. Biological pathways were ranked by decreasing *p* value and enrichment score; dotted line represents significance threshold (adjusted *p* value < 0.05).

Supplementary Figure S2 Functional analyses of differentially expressed genes in patients with poor treatment outcomes. KEGG, Reactome and Gene ontology (GO) terms and pathways enriched in DEG in patients with poor treatment outcomes. (A) KEGG (B) Reactome (C) GO biological processes and (D) GO molecular functions enriched in DEGs. All pathways were ranked by decreasing *p* value and enrichment score; dotted line represents significance threshold (adjusted *p* value < 0.05).

Supplementary Figure S3 Heatmap of gene set that differentiate slow and fast responders. No genes met the significance threshold (adjusted *p* < 0.05; fold change ≥±1.5). Green rows indicate genes with positive fold change; purple rows indicate negative fold change for slow vs fast comparison. Columns: slow responders (*n* = 29, blue) and fast responders (*n* = 30, orange).
